# LncRNA BCAR4 promotes colon cancer progression via activating Wnt/β-catenin signaling

**DOI:** 10.18632/oncotarget.21590

**Published:** 2017-10-06

**Authors:** Shurui Ouyang, Xinbin Zheng, Xin Zhou, Zhengquan Chen, Xuefeng Yang, Ming Xie

**Affiliations:** ^1^ Gastrointestinal Department, Affiliated Hospital of Zunyi Medical College, Guizhou 563000, China

**Keywords:** BCAR4, colon cancer, proliferation, Wnt/β-catenin

## Abstract

BCAR4 (Breast Cancer Anti-Estrogen Resistance 4) is a long noncoding RNA that was identified as an oncogene in breast cancer. In our research, we found that the expression level of BCAR4 was upregulated in colon cancer tissues compared to paired normal tissues. What's more, higher BCAR4 expression was correlated with lower survival rate in patients with colon cancer. Mechanistically, we showed that BCAR4 activated Wnt/β-catenin signaling in colon cancer by protecting β-catenin from degradation. We also showed that BCAR4 overexpression promoted cell proliferation and migration in colon cancer. However, silencing BCAR4 inhibited cell growth and promoted apoptosis. Besides, BCAR4 knockdown decreased tumor growth *in vivo*. These findings indicate that BCAR4 facilitated colon cancer progression by enhancing cell proliferation and inhibiting apoptosis via BCAR4/β-catenin axis. BCAR4 may be a useful new target for treatment of patients with colon cancer.

## INTRODUCTION

Colon cancer (CC) is one of the most common cancers around the world, which leads to large amounts of deaths every year [1, 2]. Up to now, there is no effective treatment available for metastatic CC. Therefore, colon cancer is still a major risk for people's health. Expression dysregulation of genes including long noncoding RNAs (lncRNA) is tightly correlated with the initiation and progression of colon cancer, which leads to some changes of biological characteristics in cancer cells, such as proliferation, migration, apoptosis and metabolism [3, 4]. Nevertheless, the underlying molecular mechanism remains elusive and urgently need to be demonstrated.

Long noncoding RNAs are transcripts of more than 200 nt in length and have no coding potential [5]. LncRNAs have been demonstrated to be of great biological functions involved in embryo development, cancer and so on [6, 7]. In mechanism, by cooperating with transcriptional factors (TFs) or remodeling complex, lncRNAs participate in regulation of gene expression [8, 9]. Besides, lncRNAs can also regulate protein stability [10]. Recently, BCAR4 (Breast Cancer Anti-Estrogen Resistance 4) that was categorized as lncRNAs was identified as an oncogene in breast cancer [11, 12]. BCAR4 can override tamoxifen-induced proliferation suppression in breast cancer [13]. What's more, van Agthoven T et al found that BCAR4 promotes proliferation of IPH-926 lobular carcinoma cells [14]. Recently, BCAR4 was shown to contribute to osteosarcoma progression via activation of GLI2-dependent gene transcription [15, 16] while it promotes chondrosarcoma cell proliferation and migration by activating mTOR signaling [17]. Additionally, upregulated expression of BCAR4 is positively correlated with poor prognosis in patients with non-small cell lung cancer [18]. However, whether BCAR4 plays a critical role in colon cancer remains to be elaborated.

In this study, we revealed that the expression levels of BCAR4 were upregulated in colon cancer tissues compared to adjacent normal tissues. Overexpressing BCAR4 in colon cancer cell line HCT8 and SW480 promotes cell proliferation and migration while BCAR4-silenced cells showed enhanced apoptosis and impaired proliferation. In mechanism, BCAR4 interacts with β-catenin to prevent its degradation. Then more activation of Wnt/β-catenin promotes cell proliferation and migration. Collectively, our results reveal a new function of BCAR4 in colon cancer and may provide a new sight on treatment of patients with colon cancer.

## RESULTS

### BCAR4 is highly expressed in colon cancer and positively correlates with poor survival rate

**Figure 1:**
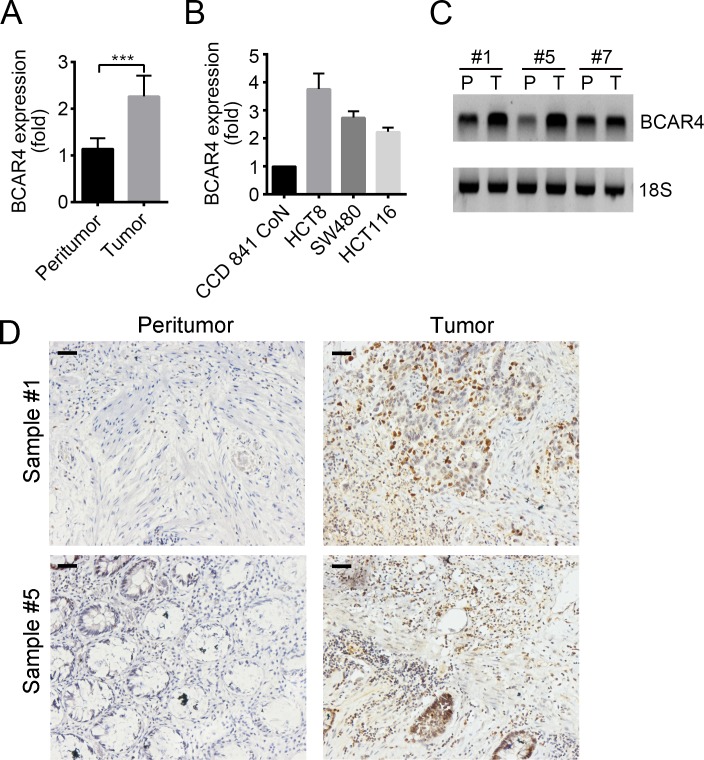
BCAR4 is highly expressed in colon cancer **(A)** Total RNAs were extracted from 20 pairs of human CC samples with Trizol. Expression levels of *BCAR4* in tumor and adjacent normal tissues were analyzed by RT-qPCR. Fold changes were normalized to endogenous *ACTB*. **(B)** Expression levels of *BCAR4* were checked in colon cancer cell lines (HCT8, SW480 and HCT116) and CCD 841 CoN cells by RT-qPCR. Fold changes were normalized to endogenous *ACTB*. **(C)** Expression levels of *BCAR4* in peritumor and CC samples were examined by Northern blot. 18S was chosen for loading control. Probes were labeled with Biotin. **(D)** BCAR4 expression in peritumor and CC samples was measured by RNA hybridization *in situ* with biotin-labeled BCAR4 probes. Scale bars, 100 μm. ^***^P<0.001 by two-tailed Student's *t* test. All data presented are shown as means ± SD collected from three independent experiments.

BCAR4 has been shown to act as an oncogene. To understand the role of BCAR4 in colon cancer, we analyzed its expression patterns. We checked the mRNA levels of BCAR4 in 20 pairs of tumor and adjacent normal tissues by RT-qPCR, and found that BCAR4 was upregulated in tumor tissues (Figure 1A). Moreover, BCAR4 was expressed higher in colon cancer cell lines such as HCT8, SW480 and HCT116 cells relative to normal human colon epithelial cell CCD 841 CoN (Figure 1B). Then we evaluated the expression level of BCAR4 by Northern blot and RNA hybridization *in situ*, and found that more BCAR4 existed in colon cancer tissues than in adjacent normal tissues (Figure 1C and 1D).

**Figure 2:**
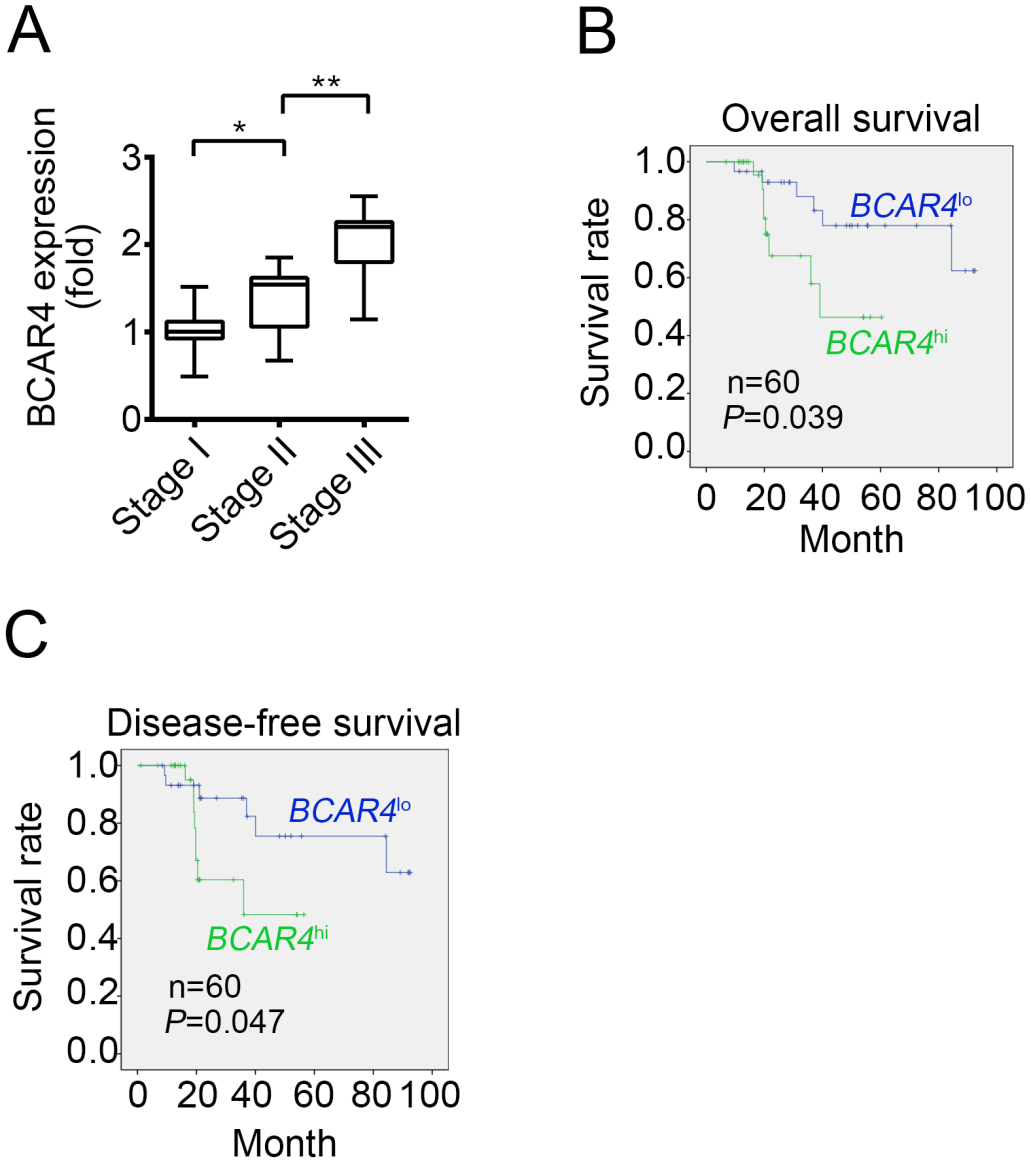
High BCAR4 expression correlates with a poor survival rate **(A)** Thirty CC samples were divided into 3 groups of Stage I, Stage II and Stage III. Then the expression levels of BCAR4 were examined in each group by RT-qPCR. Fold changes were normalized to endogenous *ACTB*. **(B** and **C)** Sixty CC samples were divided into two groups based on their expression levels. Then overall-survival rate (B) and disease-free survival rate (C) were analyzed by Kaplan–Meier survival analysis. ^*^P<0.05 and ^**^P<0.01 by two-tailed Student's t test. All data were collected from three independent experiments.

Next, we classified the cancer tissues into 3 groups of Stage I, Stage II and Stage III. As shown, BCAR4 has higher expression levels in Stage II and Stage III tissues than in Stage I tissues (Figure 2A). Then we divided 60 colon cancer samples into two groups based on BCAR4's expression levels and analyzed the overall survival rate and disease-free survival rate. We found that patients with higher expression of BCAR4 had lower survival rates, and vice versa (Figure 2B and 2C). Summarily, BCAR4 was upregulated in colon cancer and positively correlated with clinical severity and poor prognosis.

### BCAR4 promotes cell proliferation and migration

**Figure 3:**
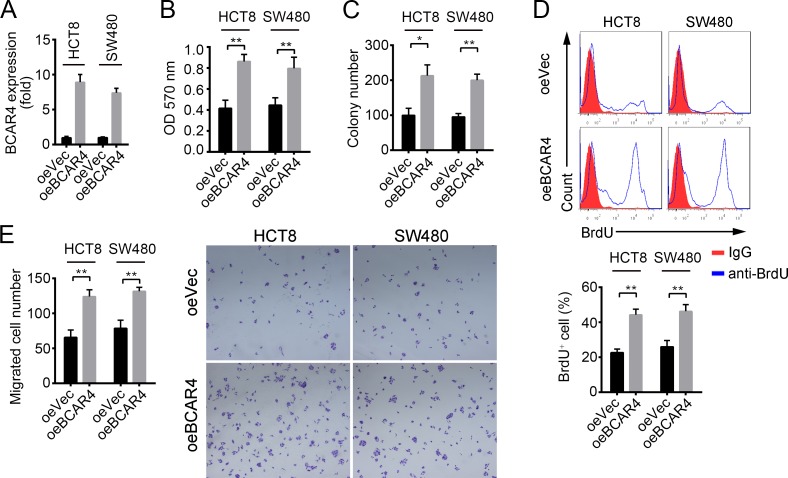
BCAR4 promotes cell proliferation and migration in CC **(A)** Overexpression of BCAR4 was confirmed by RT-qPCR in HCT8 and SW480 cells. Fold changes were normalized to endogenous *ACTB*. **(B** and **C)** Cell proliferation ability was analyzed by crystal violet assay and colony formation assays in BCAR4 overexpressing HCT8 and SW480 cells. **(D)** BCAR4 overexpression promotes cells entry into cell cycle. BrdU was added into cell medium and incubated for 2 hours in 1:1000 concentrations. Then, cells were collected, fixed and stained with anti-BrdU. BrdU^+^ cells were measured by FACS. **(E)** Overexpression of BCAR4 promotes cell migration. ^*^P<0.05 and ^**^P<0.01 by two-tailed Student's t test. All data presented are shown as means ± SD collected from three independent experiments.

To determine how BCAR4 regulates colon cancer cells, we constructed BCAR4 overexpressing plasmid by cloning BCAR4 full-length into PCDNA3 vector. We firstly confirmed that BCAR4 was overexpressed in HCT8 and SW480 cells (Figure 3A). Overexpression of BCAR4 in HCT8 and SW480 cells promoted cell growth in the crystal violet assay (Figure 3B). Also, forced expression of BCAR4 enhanced colony formation (Figure 3C). In consistent with the observations above, overexpressing BCAR4 made more cells enter into cell cycle as shown by BrdU incorporation assays (Figure 3D), suggesting that BCAR4 promotes cell proliferation in colon cancer. Moreover, enhanced expression of BCAR4 promoted migration of HCT8 and SW480 cells as shown by the Boyden chamber assay (Figure 3E). Collectively, BCAR4 promotes the proliferation and migration of colon cancer cells.

### Knockdown of BCAR4 inhibits cell proliferation and induces apoptosis

**Figure 4:**
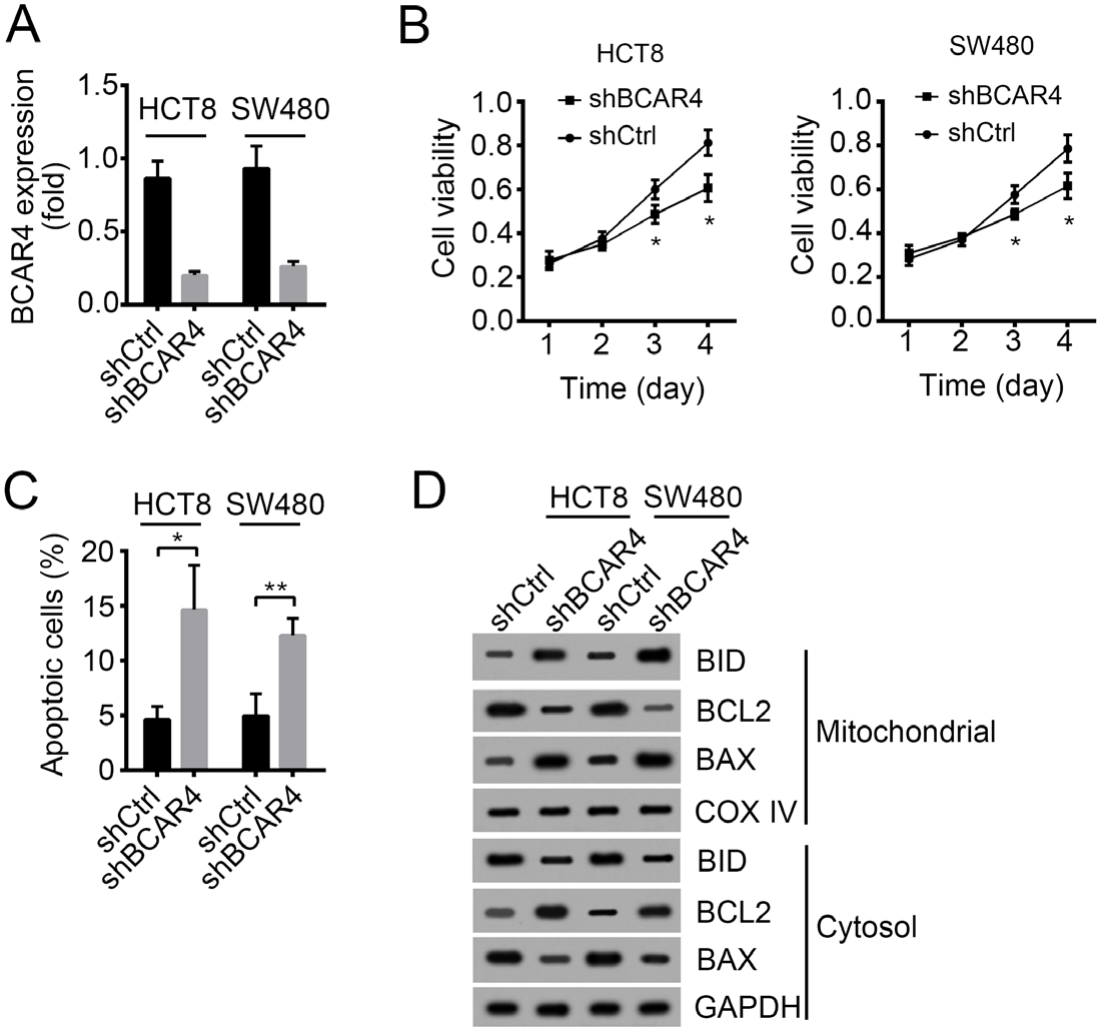
Knockdown of BCAR4 inhibits cell proliferation and induces apoptosis **(A)** The silencing efficiency of BCAR4 in HCT8 and SW480 cells was checked by RT-qPCR. Fold changes were normalized to endogenous *ACTB*. **(B)** Cell proliferation was examined by MTT assays. **(C)** Cell apoptosis was checked by Annexin V/PI staining. **(D)** BCAR4 knockdown promoted cell apoptosis as shown by Western blot. More BAX and BID appeared in mitochondrial. COX IV and GAPDH were chosen for loading control. ^*^P<0.05 and ^**^P<0.01 by two-tailed Student's t test. All data presented are shown as means ± SD collected from three independent experiments.

To further define the role of BCAR4 in colon cancer, we knocked down BCAR4 in HCT8 and SW480 cells (Figure 4A). BCAR4-silenced cells showed decreased proliferation potential as demonstrated by MTT assays (Figure 4B). What's more, BCAR4-silenced cells showed an impaired migration potential (Supplementary Figure 1A). Then we analyzed the apoptosis in HCT8 and SW480 cells after BCAR4 depletion. We found that knocking down BCAR4 promoted cell apoptosis by analysis with Annexin V/PI (Figure 4C). Additionally, after BCAR4 knocked down, BAX and BID were translocated from cytosol to mitochondrial while BCL2 were translocated from mitochondrial to cytosol, which demonstrated that BCAR4 silence promotes cell apoptosis (Figure 4D).

### BCAR4 activates Wnt/β-catenin signaling

**Figure 5:**
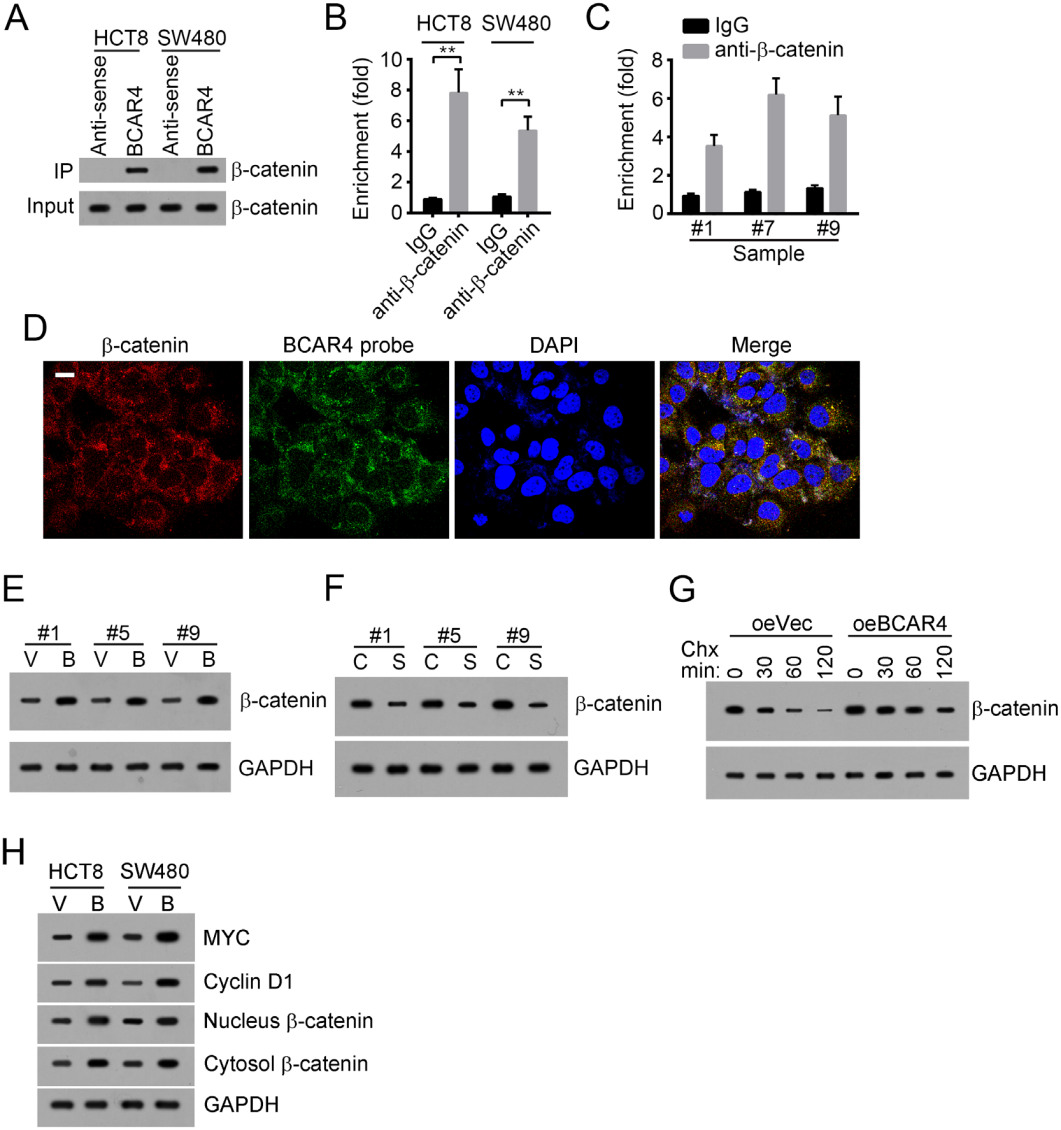
BCAR4 activates Wnt/β-catenin signaling **(A)** BCAR4 interacted with β-catenin as shown by RNA pulldown. Biotin-labeled BCAR4 was incubated with HCT8 or SW480 cell lysates, followed by SDS-PAGE and Western blot. **(B)** β-catenin enriched BCAR4 in HCT8 and SW480 cells. Anti-β-catenin was added into HCT8 or SW480 cell lysates. Then enriched RNAs were eluted and extracted. RT-qPCR was used for analysis of BCAR enrichment. **(C)** β-catenin enriched BCAR4 in CC sample cells as shown by RNA IP. **(D)** BCAR4 was colocalized with β-catenin in CC sample cells as shown by RNA FISH. BCAR4 probe was biotin-labeled. Red, β-catenin; Green, BCAR4 probe; Nuclei were stained by DAPI. Scar bar, 10 μm. **(E)** BCAR4 overexpression prevented β-catenin from degradation in CC sample cells. V, oeVector; B, oeBCAR4. **(F)** BCAR4 knockdown promoted β-catenin degradation in CC sample cells. C, shCtrl; S, shBCAR4. **(G)** BCAR4 overexpression prevented β-catenin from degradation in HCT8 cells. After CHX addition, cells were collected at indicative time points and lysed, followed by SDS-PAGE and WB. GAPDH was chosen as loading control. **(H)** BCAR4 overexpression promoted β-catenin entry into nucleus and activation of the Wnt/β-catenin signaling pathway. V, oeVector; B, oeBCAR4. ^**^P<0.01 by two-tailed Student's t test. All data presented are shown as means ± SD collected from three independent experiments.

To explore the mechanism by which BCAR4 regulates colon cancer cell, we analyzed the changes of some tumor-related signaling pathways including NK-κB signaling pathway, Notch signaling pathway, Hedgehog signaling pathway and Wnt/β-catenin signaling pathway after silencing BCAR4. We found that only Wnt/β-catenin signaling pathway was downregulated by BCAR4 depletion (Supplementary Figure 1B). Accumulating evidences showed that Wnt/β-catenin signaling was involved in the regulation of cell proliferation and migration in colon cancer [19–22]. Increased β-catenin level in intestinal cells leads to activation of Wnt/β-catenin signaling, followed by occurrence of colon cancer, which was demonstrated by APC-mutation mouse model [23]. To validate that whether BCAR4 promotes colon cancer cell proliferation by Wnt/β-catenin signaling, we first examined the interaction between BCAR4 and β-catenin by RNA pulldown and RNA IP assays. We found that biotin-labeled BCAR4 can interact with β-catenin in HCT8 and SW480 cells (Figure 5A). What's more, β-catenin also enriched BCAR4 in HCT8 and SW480 cells (Figure 5B). Additionally, β-catenin can enrich BCAR4 in colon cancer samples (Figure 5C). RNA-FISH also showed that BCAR4 was colocalized with β-catenin in CC sample cells (Figure 5D and Supplementary Figure 1C). Next, we examined how BCAR4 regulates β-catenin. We found that overexpressing BCAR4 decreased the degradation of β-catenin in colon cancer samples while knocking down BCAR4 increased the instability of β-catenin (Figure 5E and 5F). Then we added CHX into WT or BCAR4-overexpressing HCT8 cells and collected cells at indicative time points, followed by WB. BCAR4 overexpression also prevented β-catenin from degradation in HCT8 cells (Figure 5G). What's more, overexpressing BCAR4 promoted β-catenin to enter into nucleus and activated Wnt/β-catenin signaling pathway (Figure 5H). To further demonstrate that BCAR4 can activate Wnt/β-catenin signaling, we conducted a luciferase reporter assay. We cloned the promoter (-2000∼0 bp from TSS) of *MYC*, a target gene of Wnt/β-catenin signaling, to pGL3 plasmid. Overexpressing BCAR4 also activated *MYC* transcription (Supplementary Figure 1D). Previous study showed that β-catenin phosphorylation destabilized itself by promoting ubiquitination-regulated degradation [10]. We found that overexpressing BCAR4 inhibited β-catenin phosphorylation, which may then prevent its ubiquitination (Supplementary Figure 1E). In sum, BCAR4 associated with β-catenin to prevent its degradation and then activates Wnt/β-catenin signaling pathway.

### BCAR4 associates with β-catenin depending on region of nt 1000-1314

**Figure 6:**
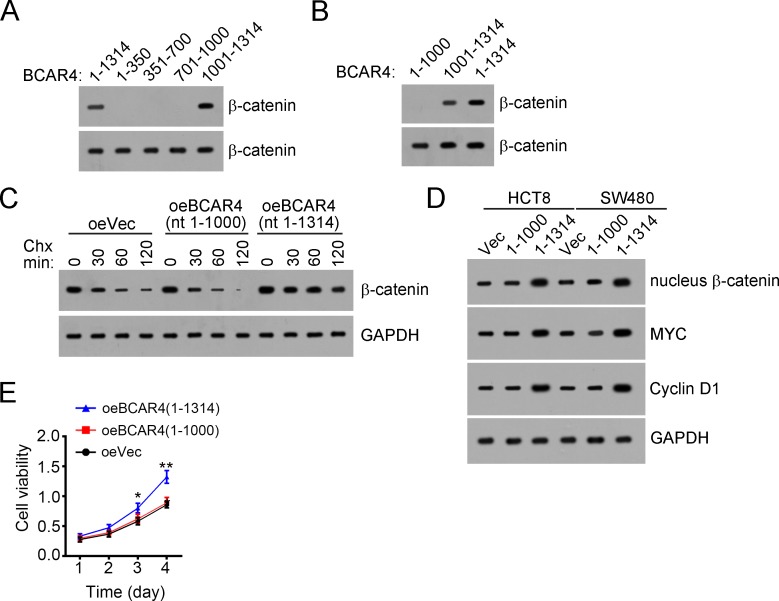
BCAR4 associates with β-catenin depending on nt 1000-1314 **(A** and **B)** BCAR4 (nt 1000-1314) interacted with β-catenin as shown by RNA pulldown assays. Biotin-labeled BCAR4 or control was added into CC sample lysates. **(C)** BCAR4 (nt 1000-1314) was essential for its function of preventing β-catenin from degradation. BCAR4 was overexpressed in HCT8 cells. **(D)** BCAR4 (nt 1000-1314) was essential for activation of Wnt/β-catenin signaling. **(E)** Deletion of nt 1000-1314 in BCAR4 impaired its potential to promote proliferation of colon cancer cells. HCT8 cells were transfected with BCAR4 or Vector and used for MTT assays. ^*^P<0.05 and ^**^P<0.01 by two-tailed Student's t test. All data presented are shown as means ± SD collected from three independent experiments.

To further validate that the interaction of BCAR4 with β-catenin is essential for the proliferation of colon cancer cells, we explored the interactive region in BCAR4 by RNA pulldown assays. We found that BCAR4 (nt 1000-1314) associated β-catenin, and deletion of nt 1000-1314 in BCAR4 abrogated its interaction with β-catenin (Figure 6A and 6B). Then we overexpressed BCAR4 (nt 1-1000) and found that deletion of nt 1000-1314 impaired the ability of BCAR4 to prevent β-catenin from degradation (Figure 6C). Besides, BCAR4 (nt 1-1000) also lose the potential to activate Wnt/β-catenin signaling (Figure 6D). Moreover, BCAR4 (nt 1-1000) cannot promote cell proliferation in CC (Figure 6E). Altogether, BCAR4 (nt 1000-1314) associated with β-catenin and was necessary for its function.

### Clinical correlation of BCAR4 expression with activation of Wnt/β-catenin signaling in CC

**Figure 7:**
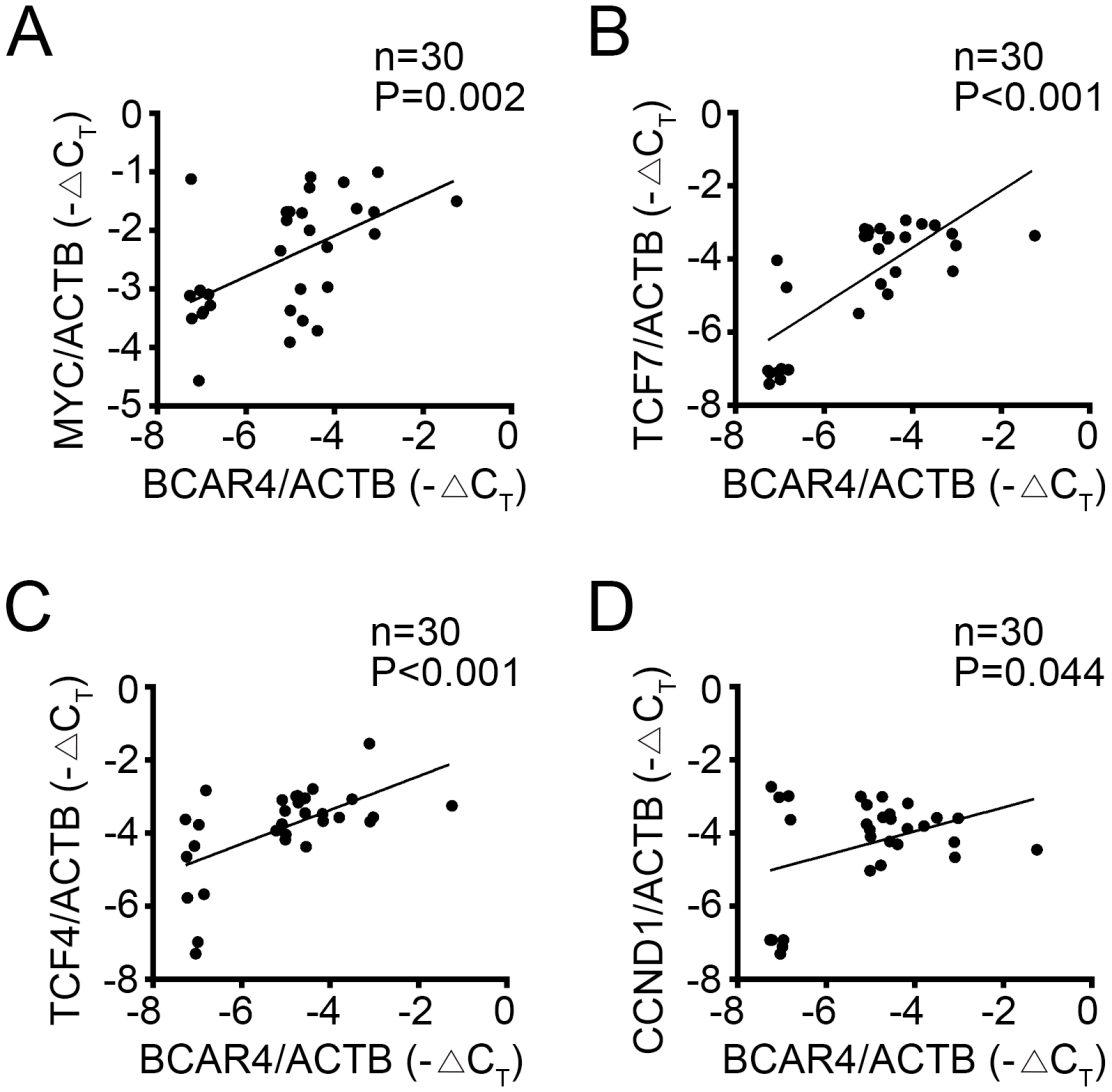
Clinical correlation of BCAR4 and activation of Wnt/β-catenin signaling in CC **(A-D)** 30 pairs of CC samples were collected and total RNA was extracted. The expression levels of MYC, TCF7, TCF4, CCND1 and BCAR4 were examined. Then the correlations of BCAR4 expression with expression of target genes of Wnt/β-catenin signaling pathway were analyzed.

To further confirm the relationship between BCAR4 and Wnt/β-catenin signaling, we detected the expression levels of BCAR4 and target genes of Wnt/β-catenin signaling by RT-qPCR in 30 colon cancer tissues. We found that BCAR4 was positively correlated with the expression levels of these target genes of Wnt/β-catenin signaling (Figure 7A-7D), which suggested that BCAR4 contributed to activation of Wnt/β-catenin signaling in CC at least in part.

### Silencing BCAR4 reduces tumor growth *in vivo*

**Figure 8:**
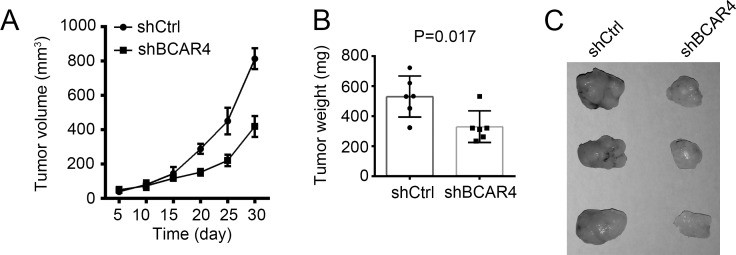
Silencing BCAR4 reduces tumor growth *in vivo* **(A)** 2×10^6^ BCAR4-depleted or control cells were injected into nude mice. The volumes of tumors were calculated at indicative time points. BCAR4 depletion significantly impaired the rate of tumor growth *in vivo*. **(B** and **C)** Tumor weight was measured on week 4 post injection.

To further examine whether BCAR4 suppressed CC tumor growth *in vivo*, we inoculated WT or BCAR4-silenced HCT8 cells into nude mice. 4 weeks later, the weights of formed tumors was measured. We found that WT HCT8 cells formed larger and heavier tumors than BCAR4-silenced cells (Figure 8A-8C). Moreover, Wnt/β-catenin signaling was downregulated in tumor tissues of nude mice derived from BCAR4-silenced HCT8 cells (Supplementary Figure 1F). Together, above findings indicated that silencing BCAR4 suppressed tumor growth *in vivo*. In sum, BCAR4 promotes tumor cell growth *in vitro* and *in vivo* by activating Wnt/β-catenin signaling. BCAR4 expression was correlated with clinical severity and prognosis in colon cancer. And BCAR4 may serve as a new target for treatment of patients with colon cancer.

## DISCUSSION

Developing novel and effective therapies for colon cancer is an urgent need now, before which we need understand the underlying mechanisms of CC development and progression better. BCAR4 expression is upregulated and acts as an oncogenic noncoding RNA in breast cancer [24, 25], non-small cell lung cancer [18] and so on. In our study, we find for the first time that BCAR4 is a key factor in colon cancer cell proliferation and migration. We show that BCAR4 was upregulated in colon cancer tissues compared to adjacent normal tissues. Overexpression of BCAR4 promotes cell proliferation and migration while its downregulation inhibits cell proliferation and increases cell apoptosis. In mechanism, we demonstrate that BCAR4 can stabilize β-catenin and activate Wnt/β-catenin signaling pathway. BCAR4 may act as a potential biomarker for diagnosis of colon cancer. And this novel BCAR4/Wnt/β-catenin axis may be useful to develop new strategies for treatment of patients with CC.

More and more evidences showed that lncRNAs play important roles in tumorigenesis and cancer progression [26–29]. They act as tumor suppressors or oncogenes through many kinds of mechanisms, including epigenetic regulation, transcriptional regulation and posttranslational regulation [10, 30, 31]. LncRNAs has been reported to participate in the regulation of tumor cell proliferation, apoptosis, migration and invasion [32–35]. Large amounts of studies have been performed about the function of lncRNAs in tumor. However, because of the specific expression patterns of lncRNAs in tissues, the relationship of dysregulation of lncRNAs with colon cancer progression is largely unknown. It is necessary to define the function of lncRNA in CC development. Our finding shows that BCAR4 is highly expressed in colon cancer and regulates cell proliferation and migration.

Wnt/β-catenin signaling is an evolutionarily conserved pathway and involved in embryonic development, tissue homeostasis and large amounts of human diseases. For example, abnormal activation of Wnt/β-catenin signaling pathway often leads to the genesis of colon cancer [36, 37]. Many groups showed that addition of Wnt/β-catenin signaling inhibitors can suppress tumor growth in colon cancer and some of the inhibitors have the potential for treatment of colon cancer [38–40]. The activity regulation on β-catenin in cancer is acknowledged as therapeutic opportunities [41]. Nevertheless, how the Wnt/β-catenin signaling pathway is regulated delicately remains to be defined. A recent reports shows that KDM3 controls human colorectal cancer stem cells by regulating Wnt target gene transcription through epigenetic modification [42]. Another research shows that lnc-β-Catm regulates β-catenin stabilization and sustains liver CSC self-renewal [10]. However, knowledge about the regulation of β-catenin stabilization in colon cancer is limited. In this report, we showed that BCAR4 interacts with β-catenin and prevents its degradation, which leads to more activated β-catenin existed in the nucleus.

In summary, our study showed that BCAR4 was highly expressed in CC and promoted cell proliferation and migration via activation of Wnt/β-catenin signaling pathway. This finding suggests that BCAR4 may serve as a novel biomarker in CC and a potential therapeutic target for treatment of colon cancer.

## MATERIALS AND METHODS

### Patient samples

Sixty pairs of CC tissues and adjacent normal tissues involved in our study were collected from Affiliated Hospital of Zunyi Medical College. Written consents to approve our use of these tissues in the research were obtained from all patients. The protocol was approved by Affiliated Hospital of Zunyi Medical College. All methods involving human patients were performed in accordance with the relevant guidelines and regulations of Affiliated Hospital of Zunyi Medical College.

### Cell lines and cell culture

Human colon cancer cell lines (HCT8, SW480 and HCT116) were obtained from ATCC and maintained in DMEM medium supplemented with 10% fetal bovine serum (FBS; Invitrogen), 100 μg/ml penicillin and 100U/ml streptomycin. Normal human colon epithelial cells CCD 841 CoN (ATCC) were cultured in Eagle's Minimum Essential Medium and supplemented with 10% FBS. Cells were incubated at 37°C in a humidified atmosphere with 5% CO_2_.

### Cell transfection

BCAR4 full-length was cloned into pCDNA3 plasmid. shBCAR4 (5′-GGGACTTGAGTTATGTTGGTGGCTA-3′) was synthetized by invitrogen and cloned into pGPH1/Neo (GenePharma, Shanghai, China) as described before [15]. BCAR4 overexpression was achieved by transfecting pCDNA3-BCAR4 into HCT8 and SW480 cells with Lipofectamine 3000 (Invitrogen, Carlsbad, CA, USA). pGPH1-shBCAR4 or control plasmid was transfected into HCT8 or SW480 cell using Lipofectamine 3000 and selected with neomycin (1000 μg/ml) for 4 weeks.

### Antibodies

Anti-BCL2 (4223), anti-BID (8762), anti-BAX (5023), anti-COX IV (4850), anti-GAPDH (5014), anti-β-catenin (9562), anti-phospho-β-catenin (T41/S45, 9565), anti-MYC (9402) and anti-CYCLIN D1 (2922) were purchased from Cell Signaling Technology.

### Apoptosis analysis

Apoptosis analysis was performed through Annexin V-FITC/PI apoptosis detection kit (eBiosciences) by FACS.

### Xenograft tumor formation

We purchased six-week-old male BALB/c nude mice from HFK Biosciences and maintained under pathogen-free conditions with approval by Affiliated Hospital of Zunyi Medical College. For tumor propagation analysis, 2×10^6^ *BCAR4*-silenced cells were subcutaneously injected into BALB/c nude mice. Tumor weight was measured on week 5 post injection. Animal experiments were performed in accordance with relevant guidelines and regulations of the Institutional Animal Care and Use Committees at Affiliated Hospital of Zunyi Medical College, and protocols were approved by the Institutional Animal Care and Use Committees at Affiliated Hospital of Zunyi Medical College.

### MTT, colony formation and migration assays

For MTT assays, 1×10^3^ cells were seeded into 96-well plates. Cell proliferation was measured at indicative time points. MTT (20 μl, 5 mg/ml) (Sigma, USA) was added into each well and incubated for 4 h at 37°C. Then 150 μl DMSO was added to solubilize the crystals. To determine cell viability, the absorbance was read at wavelength of 570 nm and 650 nm (background reading subtracted) with a microplate reader (Bio-rad).

For colony formation assays, 2×10^3^ cells were seeded into a 6-well plate and incubated at 37°C for 12 days. And then the cells were fixed in 90% ethanol and stained with crystal violet solution. The formed colonies were counted.

For cell Migration assays, the transwell filter chambers (Costar, Corning, NY) were used according to the manufacturers’ instructions. Briefly, 2×10^5^ cells were resuspended in serum-free medium and added into the top chamber. Medium containing 10% FBS was added to the lower chamber. After incubation for 12 h, cells on the lower surface were stained, photographed, and counted by a microscope in six random fields for each group.

### Real-time quantitative PCR

Total RNAs were extracted with TRIzol according to the manufacturer's protocol. Then cDNA was synthesized with the M-MLV reverse transcriptase (Promega). Then mRNA transcripts were analyzed with ABI 7300 qPCR system using specific primer pairs. Relative expressions were calculated and normalized to endogenous *Actb*. The primer sequence information is available if requested.

### Northern blot

Protocols for Northern blot have been described before [9]. In brief, total RNA was extracted from sample cell with TRIzol. 10 μg RNA from each sample was subjected to formaldehyde-denaturing agarose electrophoresis followed by transferring to positively charged NC film with 20×SSC buffe. Membrane was UV cross-linked and incubated with hybrid buffer for a 2 h prehybridization, followed by incubation with biotin-labeled RNA probes (BCAR4: nt40∼280). Biotin signals were detected with HRP-conjugated streptavidin according to the manufacturer's instruction.

### *In situ* hybridization

CC and peri-tumour samples were fixed and embedded with paraffin. Then sample sections were incubated in graded alcohols and incubated in 3% hydrogen peroxide (H_2_O_2_) for 30 min. Biotin-conjugated probes and streptavidin-HRP conjugate were used for ISH. The samples were finally stained with haematoxylin.

### RNA pulldown

Cells were lysed with RIPA lysis buffer and biotin-labeled BCAR4 was added for incubation at 4°C overnight. Then Streptavidin-magnetic C1 beads were added and incubated at 4°C for 2h. Then Streptavidin-magnetic C1 beads were collected and IP components were separated by SDS-PAGE, followed by WB.

### RNA FISH

Fluorescence-conjugated BCAR4 probes were generated according to protocols from Biosearch Technologies and synthesized by Invitrogen. Sequences of probes were as follows: 5′-GGACGACAGGACGACTTCTA-3′; 5′-CGGTAACGATGTTACTGTGA-3′; 5′-CTGGTT GGCTTTCAGTTGAC-3′. CC samples were hybridized with DNA probe sets and then immune-stained with anti-β-catenin. Images were obtained with Olympus FV1200 laser scanning confocal microscopy (Olympus).

### Statistical analysis

All statistical analyses were performed using the Statistical Package for the Social Sciences version 20.0 software (SPSS Inc., Chicago, IL, USA). Survival curves were calculated using the Kaplan-Meier method and were analyzed using the log-rank test. For comparisons, one-way analyses of variance and two-tailed Student's t-tests were performed, as appropriate. *P*< 0.05 was considered statistically significant.

## SUPPLEMENTARY MATERIALS FIGURE


