# Supplementary material for: LncRNA BCAR4 promotes colon cancer progression via activating Wnt/β-catenin signaling
Source: Oncotarget. 2017 Oct 6;8(54):92815–26. doi: 10.18632/oncotarget.21590 (PMC5696224; doi:10.18632/oncotarget.21590)
Supplement: Supplementary file 1 [file oncotarget-08-92815-s001.pdf]

LncRNA BCAR4 promotes colon cancer progression via activating Wnt/β-catenin signaling

SUPPLEMENTARY MATERIALS

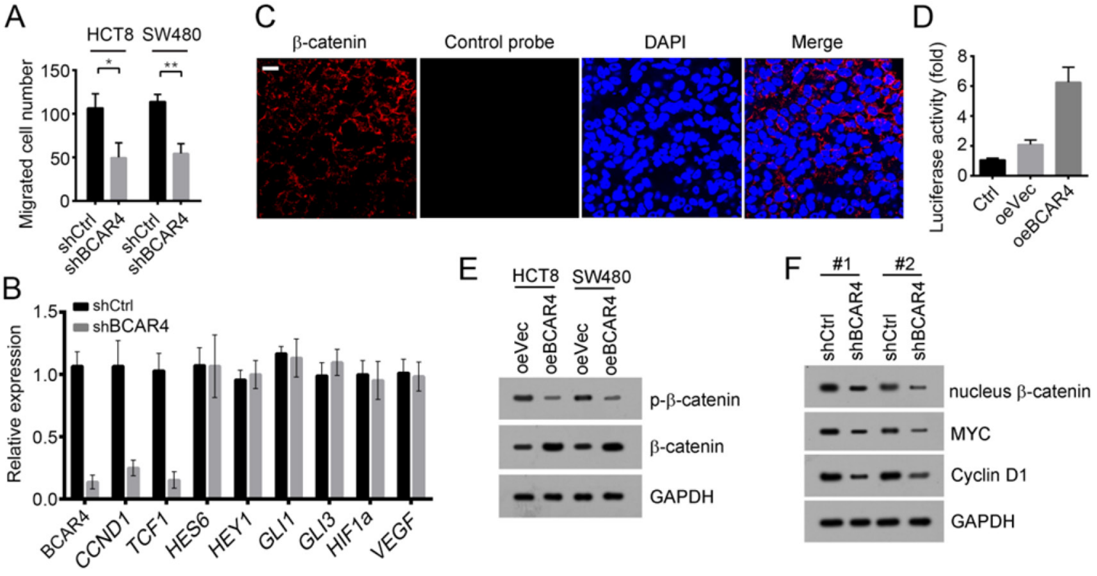

Supplementary Figure 1: BCAR4 activates Wnt/β-catenin signaling. **(A)** BCAR4-depleted cells showed decreased migration potential. **(B)** BCAR4 knockdown impaired Wnt/β-catenin signaling in CC cells. Total RNAs were extracted from WT or BCAR4-silenced HCT8 cells. Fold changes were normalized to endogenous *ACTB*. **(C)** β-catenin colocalized with BCAR4 in CC sample cells as shown by RNA FISH. Red, β-catenin; Green, Control probe; Nuclei were stained by DAPI. Scar bar, 10 μm. **(D)** pGL3-MYC and BCAR4 overexpressing plasmid or empty control were transfected into cells. 24 h later, luciferase activity was measured. **(E)** Overexpressing BCAR4 inhibited β-catenin phosphorylation. **(F)** BCAR4 depletion downregulated Wnt/β-catenin signaling in tumor tissues of nude mice. $^{*}P<0.05$ and $^{**}P<0.01$ by two-tailed Student's t test. All data presented are shown as means ± SD collected from three independent experiments.
